# Special Issue “Translating Molecular Psychiatry: From Biomarkers to Personalized Therapies”

**DOI:** 10.3390/ijms262010238

**Published:** 2025-10-21

**Authors:** Masaru Tanaka

**Affiliations:** Danube Neuroscience Research Laboratory, HUN-REN-SZTE Neuroscience Research Group, Hungarian Research Network, University of Szeged (HUN-REN-SZTE), Tisza Lajos krt. 113, H-6725 Szeged, Hungary; tanaka.masaru.1@med.u-szeged.hu; Tel.: +36-62-342-847

## 1. Introduction: Psychiatry at the Molecular Crossroads

Psychiatry stands at a turning point, where molecular insights promise to revolutionize how we diagnose, monitor, and treat neuropsychiatric conditions, including Alzheimer’s, Parkinson’s, depression, dementia, and schizophrenia (SCZ), among others. For decades, diagnoses have relied on descriptive syndromes, while treatment decisions have been guided by trial and error rather than molecularly grounded evidence [1,2,3,4,5,6]. Despite the remarkable growth of neuroscience, few insights have crossed the bridge into clinical psychiatry [1,2,5,7,8]. Treatments for depression, bipolar disorder (BD), SCZ, and dementia remain only partially effective, and diagnostic overlap between major psychiatric conditions continues to hinder precision care [9,10,11,12,13,14].

Against this backdrop, the concept of molecular psychiatry has emerged [8,15,16,17]. Its central promise is straightforward yet ambitious: to translate biological signatures into clinical tools for diagnosis, prognosis, and therapy selection [2,15,18,19,20,21]. However, the road from bench to bedside is far from linear [2,16,17,22,23,24]. Many biomarker findings remain inconsistent, mechanistic insights often lack replication, and new therapeutic candidates are slow to reach clinical trials [2,16,25,26,27].

This Special Issue, Translating Molecular Psychiatry: From Biomarkers to Personalized Therapies (https://www.mdpi.com/journal/ijms/special_issues/H435J0IJA5 (accessed on 12 October 2025)), directly addresses these challenges. It brings together eight contributions that span the spectrum—from inflammation in mood disorders to blood–brain barrier (BBB) dysfunction in SCZ, from RNA editing biomarkers and AI-driven diagnostics to novel therapeutic approaches in AD and neurodevelopmental disorders. Collectively, these papers exemplify the current state of the field and offer a window into its future.

## 2. Mapping the Terrain: Current Insights from the Special Issue

This Special Issue brings together eight papers that highlight the ways molecular psychiatry is steadily moving toward translation, with work ranging from mood disorders and SCZ to biomarker discovery and the development of new therapies. Each contribution takes a different route, yet together they form a coherent map pointing toward a psychiatry that is at once mechanistically rigorous and clinically meaningful. Organized into four overarching themes, these studies reveal how seemingly distinct lines of research can converge on a single aim: advancing personalized care (Table 1).

### 2.1. Immune-Related Insights in Mood Disorders

Ríos et al. examined inflammatory biomarkers in 109 euthymic bipolar patients, focusing on interleukin-6 (IL-6) and high-sensitivity C-reactive protein (hs-CRP) [28]. They found that IL-6, but not hs-CRP, was linked to cognitive deficits in memory, attention, and visuospatial skills, as well as to increased hospitalization risk. This positions IL-6 as a candidate biomarker for cognitive vulnerability in BD and underscores the clinical significance of immune dysregulation. Bottaccioli et al. offered a conceptual advance, critiquing the reductionist search for a single molecular “switch” in depression [29]. Instead, they argued for a psychoneuroendocrineimmunology (PNEI) model, which views depression as a systemic disorder shaped by psychological, biological, and behavioral interactions. Their call for integrated, personalized interventions reframes depression as a multifaceted condition requiring multidimensional care (Table 1).

### 2.2. Schizophrenia (SCZ): Treatment Optimization and BBB Dysfunction

Trovini et al. compared two strategies for initiating long-acting injectable (LAI) aripiprazole in 152 patients [30]. Both regimens improved psychopathology, but the two-injection start (TIS) maintained therapeutic serum levels without the high peaks seen in the one-injection start (OIS), suggesting a safer pharmacokinetic profile. This practical study provides immediate guidance for optimizing SCZ treatment. Zhang et al. reviewed evidence that BBB dysfunction plays a central role in SCZ pathophysiology [31]. They integrated findings from postmortem studies, biomarker assays, and advanced imaging to show how endothelial dysfunction, tight junction abnormalities, and neuroinflammation disrupt barrier integrity. Importantly, they discussed the bidirectional effects of antipsychotics on BBB function, highlighting its dual role as a therapeutic target and potential liability.

### 2.3. Precision Biomarkers and AI in Psychiatric Diagnosis

Checa-Robles et al. presented proof-of-concept evidence that RNA editing biomarkers, when analyzed with machine learning, can distinguish between BD, SCZ spectrum disorders, and healthy controls [32]. This integration of molecular data and computational tools marks a significant step toward objective, biomarker-based diagnosis in psychiatry. Șerban et al. focused on neuropsychiatric syndromes caused by brain tumors [33]. They argued that these syndromes are intrinsic manifestations of tumor biology, driven by metabolic reprogramming, cytokine release, and circuit disruption. By highlighting lesion-network mapping, exosomal biomarkers, and AI predictive modeling, they envisioned new strategies for early detection and precision treatment, reframing neuro-oncology as a frontier of psychiatric translation.

### 2.4. Novel Therapies in Alzheimer’s and Developmental Disorders

Barbalho et al. systematically reviewed studies on AdipoRon, an adiponectin receptor agonist, in AD models [34]. AdipoRon reduced inflammation, improved mitochondrial function, mitigated tau hyperphosphorylation, and enhanced cognition, suggesting a promising multi-target therapeutic. The authors identified key research gaps, emphasizing the need for translational work in humans. Ayash et al. used a rat model of Group B Streptococcus (GBS)-induced chorioamnionitis to test IL-1 receptor antagonist (IL-1Ra) [35]. Male offspring exposed in utero developed autism- and cerebral palsy (CP)-like traits, but maternal IL-1Ra treatment reversed these outcomes. The findings point to immunomodulation during pregnancy as a potential preventive strategy against infection-driven neurodevelopmental disorders.

## 3. Present Challenges and Gaps

Despite promising advances, molecular psychiatry still faces significant hurdles before translation can become reality. The eight contributions, while diverse in scope, collectively highlight recurring gaps that restrain progress. These include methodological inconsistencies, fragmented frameworks, and limited clinical validation. Such persistent obstacles underscore the pressing need for integrative strategies that bridge discovery and practice [36].

First, biomarker research in molecular psychiatry continues to struggle with problems of reproducibility and limited scale. IL-6 has been identified as a potential marker in BD, and RNA editing holds diagnostic promise [37,38,39,40,41,42]. Yet, such findings arise from modest cohorts, and without rigorous multisite validation, clinical translation remains premature [2,37,43,44,45].

Diagnostic overlap remains an enduring challenge that continues to complicate translation [43,45,46,47,48]. Biomarker discovery and AI-driven approaches have offered promising tools, yet they fall short when confronted with the blurred boundaries of psychiatric nosology. Disorders such as BD and SCZ share symptoms and molecular signatures [49,50,51,52,53]. Clear differentiation demands methodological refinement alongside conceptual innovation [48,54,55,56,57].

Barriers in clinical translation remain evident, as progress from preclinical discovery to human application proves limited [58,59]. Novel therapeutics highlight this gap. AdipoRon, for example, shows neuroprotective effects in animal models, yet its safety and efficacy in humans remain unknown [60]. Prenatal IL-1 blockade presents intriguing possibilities, but ethical, regulatory, and safety concerns restrict its immediate evaluation in clinical pregnancy trials [61,62].

Finally, psychiatry continues to wrestle with its conceptual framework. The PNEI model for depression highlights the danger of reductionism, yet systemic integration remains more aspirational than operational in current clinical settings. A similar tension exists in neuro-oncology, where psychiatric dysfunction is often treated as secondary rather than intrinsic to tumor biology.

Conceptual gaps persist as psychiatry struggles with its foundational frameworks [29,63,64]. Despite advances, reliance on reductionist models continues to overshadow systemic approaches. The PNEI model underscores the pitfalls of narrow perspectives in depression research [29,65]. In neuro-oncology, a parallel tension emerges, where psychiatric dysfunction is often regarded as secondary instead of being intrinsic to tumor biology [66].

Together, these challenges make clear that progress in molecular psychiatry requires more than incremental advances. What is needed is a deliberate integration of validated biomarkers, mechanistic insight, and carefully designed clinical trials. Only by addressing methodological, diagnostic, translational, and conceptual barriers in concert can the field truly move toward personalized and effective psychiatric care.

## 4. Building the Bridge: Advances Highlighted in This Special Issue

This collection makes a distinctive contribution by linking mechanistic discoveries with translational relevance across psychiatric and neurological domains. Although significant hurdles remain, the studies presented here demonstrate clear progress. Each contribution advances understanding in a way that pushes the field closer to practical applications, offering tangible steps toward clinical translation.

In the domain of inflammation, two contributions stand out for their translational significance. One identifies IL-6 as a predictor of cognitive impairment in BD, highlighting its role as a potential biomarker [28]. The other demonstrates how interleukin-1 (IL-1) blockade may prevent neurodevelopmental injury [35]. Together, these findings strengthen the connection between immune dysregulation and psychiatric outcomes.

Advances in treatment optimization for SCZ are illustrated by two complementary findings. Clinically, the tailored intermittent strategy for initiating LAIs enhances safety and stability, offering direct guidance for practice [30]. Mechanistically, recognition of BBB dysfunction reframes pathophysiology, opening new avenues for both diagnostic refinement and innovative therapeutic strategies [31].

Advances in biomarker precision reveal how molecular and computational tools can reshape psychiatric diagnosis. RNA editing signatures integrated with artificial intelligence (AI) provide a compelling proof-of-concept for distinguishing complex disorders with greater accuracy [32]. Equally transformative is the recognition that tumor-associated psychiatric syndromes arise intrinsically from cancer biology, forging new connections between oncology and psychiatry [33].

Finally, progress in novel therapeutics illustrates how complex disorders may benefit from multi-target strategies. AdipoRon emerges as a promising candidate, reducing neuroinflammation, mitigating tau pathology, and improving cognition in preclinical models of AD [34]. At the same time, tumor-induced psychiatric dysfunction is reframed as a primary condition, expanding therapeutic horizons.

Collectively, these advances illustrate a decisive shift in psychiatry from reliance on descriptive syndromes toward biologically anchored frameworks. What emerges is a discipline that increasingly integrates mechanistic discovery with translational potential [67,68,69,70,71]. By aligning molecular insights with clinical application, the field is steadily constructing bridges toward precision and personalized care.

## 5. The Road Ahead

The future of molecular psychiatry lies in a stepwise progression, where short-term goals provide the groundwork for larger transformations over the next decade. This trajectory can be envisioned in two phases: immediate priorities over the next two to four years and broader, paradigm-shifting advances within five to ten years.

### 5.1. Near-Term (2–4 Years)

In the near term, priorities in mood disorder research will focus on validating IL-6 as a reliable biomarker [38,72,73,74]. Multisite longitudinal studies are essential to confirm its predictive value for cognitive outcomes and clinical trajectories. Alongside this effort, small-scale clinical trials will begin to test anti-inflammatory strategies in BD, aiming to determine feasibility, safety, and preliminary efficacy [75,76,77]. Together, these initiatives could establish immune modulation as a tangible therapeutic pathway [78,79,80].

For SCZ, advances in treatment protocols and mechanistic exploration will take center stage [81,82,83]. The tailored intermittent strategy for initiating LAIs is poised to enter clinical guidelines, where its potential to enhance safety and stability could shift prescribing practices [84]. At the same time, advanced imaging methods will be deployed to systematically characterize BBB disruption [31,81,83]. Such studies may reveal how endothelial dysfunction and immune interactions contribute to disease progression, creating opportunities for therapeutic targeting [81,82,83].

Biomarker precision will advance through replication and expansion [85]. RNA editing signatures must be validated in larger, well-characterized cohorts to move from proof-of-concept toward robust application [32,86,87]. Integration with multi-omics platforms, including genomics, proteomics, and metabolomics, will further strengthen diagnostic potential by capturing the multidimensional complexity of psychiatric disorders [88,89,90]. These combined approaches could eventually yield clinically usable biomarker panels that surpass the limitations of symptom-based diagnosis.

In neurodegeneration and developmental psychiatry, early translational steps will be pursued with caution but also with urgency [90]. Pilot human studies of AdipoRon will test whether its multi-target effects observed in preclinical models can translate into meaningful outcomes in AD [34,91,92]. At the same time, early-phase trials of IL-1 blockade in high-risk pregnancies or neonatal populations will be considered, though only under rigorous ethical and safety oversight [61,93,94]. These initiatives could set the stage for transformative preventive strategies (Table 2).

### 5.2. Long-Term (5–10 Years)

In the next decade, psychiatry is likely to be transformed by AI–guided biomarker panels that combine molecular signatures, clinical features, and digital phenotyping [95,96,97,98]. These platforms will not only improve early diagnosis but also guide personalized therapy selection [8,96,99]. Such tools have the potential to move psychiatry away from trial-and-error prescribing and toward precision interventions matched to each patient’s biological and behavioral profile [8,69,96].

Alongside these advances, a systemic paradigm informed by the PNEI model will shape integrated treatment approaches [29,100,101]. Care will no longer be divided into isolated domains but will instead merge biological, psychological, and behavioral dimensions into a unified framework [29,100,102]. This integrative strategy is expected to produce durable outcomes, offering improvements that reductionist models have struggled to achieve [29,103,104].

Neuro-oncology will provide a compelling example of how these principles can be applied in practice [105,106,107]. Psychiatric dysfunction in patients with brain tumors will increasingly be recognized as an intrinsic manifestation of tumor biology [107,108,109]. In response, clinical protocols will evolve to combine oncological and psychiatric management from the outset [105,106,108]. Such integration could ensure that cognitive and affective symptoms are addressed as core features of disease, not as secondary complications [110,111].

Preventive psychiatry is also poised to expand its scope [112,113,114]. Immunomodulation during pregnancy may become a validated protective strategy against infection-driven neurodevelopmental disorders [115,116,117]. With careful ethical oversight and rigorous testing, such approaches could significantly reduce the burden of conditions like autism spectrum disorders and CP, shifting psychiatry toward a model of early intervention and prevention [116,118,119].

Finally, progress in neurodegeneration will focus on AD, where multi-target agents such as AdipoRon may enter clinical practice [34,91,120]. Coupled with biomarker-based early detection, these therapies could intervene before irreversible cognitive decline occurs [121,122,123]. Together, these innovations promise a decade in which psychiatry evolves into a discipline defined by prevention, precision, and integration [124].

Viewed as a whole, the near- and long-term trajectories suggest a field on the verge of transformation. Incremental advances will build the scaffolding for breakthroughs, while paradigm shifts will redefine how psychiatry is practiced. The road ahead is ambitious yet achievable, charting a future of precision, prevention, and integrative care.

## 6. Conclusions: From Discovery to Translation

The eight contributions of this Special Issue highlight a psychiatry in transition, shifting from descriptive diagnoses and symptom-based treatments toward mechanistically informed, biomarker-driven, and personalized care. Collectively, they demonstrate how translational research can illuminate the path forward. Immune markers such as IL-6 provide insight into cognition in BD, while AI applied to RNA editing offers a proof-of-concept for diagnostic precision. Advances in SCZ research show that optimized initiation strategies for LAIs can enhance safety, while recognition of BBB dysfunction reframes disease mechanisms. In parallel, novel therapeutics such as AdipoRon in Alzheimer’s models and prenatal IL-1 blockade in developmental contexts highlight the translational potential of multi-target and preventive approaches. Yet these strides are not achieved in isolation. The field’s future depends on sustained collaboration that brings psychiatry into conversation with neurology, immunology, oncology, and computational sciences. By weaving together these disciplines, psychiatry can transform molecular discoveries into clinically actionable strategies. Precision psychiatry is no longer a distant aspiration but an emerging reality that requires collective commitment to become fully realized.

## Figures and Tables

**Table 1:** Advances in molecular psychiatry: Immune, biomarker, and therapeutic frontiers. Summary of key insights from recent studies featured in this issue, spanning immune-related mechanisms in mood disorders, treatment strategies and blood–brain barrier (BBB) dysfunction in schizophrenia (SCZ), precision biomarker discovery through AI integration, and novel therapeutic approaches for Alzheimer’s and neurodevelopmental disorders. Together, these findings illustrate how diverse lines of inquiry are converging to refine mechanistic understanding and drive personalized psychiatry.

Category	Key Points (≤3)	Ref.
Immune-Related Insights in Mood Disorders	IL-6, not hs-CRP, linked to cognitive deficits in BD. Higher IL-6 predicted worse memory, attention, visuospatial skills. Elevated IL-6 correlated with greater hospitalization risk.	[28]
	Critiques reductionist biomarker-based models. Proposes systemic PNEI framework for depression. Advocates integrated, personalized interventions.	[29]
SCZ: Treatment Optimization and BBB Dysfunction	Compared OIS vs. TIS regimens in 152 patients. Both effective, but TIS avoided toxic serum peaks. TIS offers safer, more stable pharmacokinetics.	[30]
	Synthesized evidence of BBB disruption in SCZ. Implicated endothelial, tight junction, and immune dysfunction. BBB is both vulnerability and therapeutic target.	[31]
Precision Biomarkers and AI in Psychiatric Diagnosis	RNA editing biomarkers analyzed with machine learning. Distinguished BD, SCZ spectrum, and healthy controls. Demonstrated diagnostic potential of molecular–AI integration.	[32]
	Reviewed psychiatric syndromes from brain tumors. Highlighted metabolic, cytokine, and circuit disruptions. Proposed AI/biomarker-based neuro-oncology–psychiatry paradigm.	[33]
Novel Therapies in Alzheimer’s and Developmental Disorders	Systematic review of six preclinical studies. AdipoRon reduced neuroinflammation, tau pathology, improved cognition. Calls for translational studies in humans.	[34]
	Rat model of GBS-induced chorioamnionitis. Maternal IL-1Ra prevented autism- and CP-like traits. Suggests prenatal immunotherapy to prevent neurodevelopmental disorders.	[35]

AI, artificial intelligence; BBB, blood–brain barrier; BD, bipolar disorder; CP, cerebral palsy; GBS, group B streptococcus; hs-CRP, high-sensitivity C-reactive protein; IL-1Ra, interleukin-1 receptor antagonist; IL-6, interleukin-6; OIS, one-injection start; PNEI, psychoneuroendocrineimmunology; RNA, ribonucleic acid; SCZ, schizophrenia; TIS, two-injection start.

**Table 2:** Translational trajectories in molecular psychiatry: From current gaps to future directions. Timeline of advances across four major domains of molecular psychiatry. The table highlights persisting gaps, key contributions from this Special Issue, and projected trajectories over the near term (2–4 years) and long term (5–10 years). Together, these directions illustrate how biomarker validation, mechanistic insight, and integrative frameworks may converge to shape precision psychiatry.

Category	Key Gaps	Advances from this Special Issue	Future Directions (Near-Term 2–4 yrs/Long-Term 5–10 yrs)
Mood Disorders	Inconsistent biomarker findings. Lack of systemic treatment frameworks.	IL-6 predicts cognition in BD. PNEI paradigm reframes depression.	Near-term: Validate IL-6; test anti-inflammatory interventions. Long-term: Integrate PNEI into personalized care.
SCZ	Limited optimization of LAI protocols. Poor understanding of BBB pathology.	TIS regimen improves safety. BBB identified as mechanistic target.	Near-term: Adopt TIS in practice; imaging BBB dysfunction. Long-term: BBB-targeted therapies; personalized antipsychotics.
Precision Biomarkers & AI	Small-scale biomarker studies. Diagnostic overlap of BD and SCZ.	RNA editing + AI distinguishes conditions. Tumor syndromes reframed as intrinsic.	Near-term: Replicate RNA panels; expand AI models. Long-term: Clinical AI platforms for biomarker-based diagnosis.
Neurodegeneration & Developmental disorders	Preclinical–clinical gap. Ethical challenges in prenatal interventions.	AdipoRon shows neuroprotection. IL-1 blockade reverses prenatal injury traits.	Near-term: Pilot human studies (AdipoRon, IL-1). Long-term: Embed multi-target AD therapies; preventive immunomodulation in pregnancy.

AD, Alzheimer’s disease; AI, artificial intelligence; BBB, blood–brain barrier; BD, bipolar disorder; IL-1, interleukin-1; IL-6, interleukin-6; LAI, long-acting injectable; PNEI, psychoneuroendocrineimmune; SCZ, schizophrenia; TIS, two-injection start.

## Abbreviations

The following abbreviations are used in this manuscript:

AD	Alzheimer’s disease
AI	artificial intelligence
BBB	blood–brain barrier
BD	bipolar disorder
CP	cerebral palsy
GBS	group B streptococcus
hs-CRP	high-sensitivity C-reactive protein
IL-1	interleukin-1
IL-1Ra	interleukin-1 receptor antagonist
IL-6	interleukin-6
LAI	long-acting injectable
OIS	one-injection start
PNEI	psychoneuroendocrineimmunology
SCZ	schizophrenia
TIS	two-injection start
